# The Role of Oxidative Stress-Induced Epigenetic Alterations in Amyloid-***β*** Production in Alzheimer's Disease

**DOI:** 10.1155/2015/604658

**Published:** 2015-10-12

**Authors:** Li Zuo, Benjamin T. Hemmelgarn, Chia-Chen Chuang, Thomas M. Best

**Affiliations:** ^1^Radiologic Sciences and Respiratory Therapy Division, School of Health and Rehabilitation Sciences, The Ohio State University College of Medicine, The Ohio State University Wexner Medical Center, Columbus, OH 43210, USA; ^2^Molecular Physiology and Rehabilitation Research Laboratory, School of Health and Rehabilitation Sciences, The Ohio State University College of Medicine, The Ohio State University Wexner Medical Center, Columbus, OH 43210, USA; ^3^Division of Sports Medicine, Department of Family Medicine, Sports Health & Performance Institute, The Ohio State University Wexner Medical Center, Columbus, OH 43210, USA

## Abstract

An increasing number of studies have proposed a strong correlation between reactive oxygen species (ROS)-induced oxidative stress (OS) and the pathogenesis of Alzheimer's disease (AD). With over five million people diagnosed in the United States alone, AD is the most common type of dementia worldwide. AD includes progressive neurodegeneration, followed by memory loss and reduced cognitive ability. Characterized by the formation of amyloid-beta (A*β*) plaques as a hallmark, the connection between ROS and AD is compelling. Analyzing the ROS response of essential proteins in the amyloidogenic pathway, such as amyloid-beta precursor protein (APP) and beta-secretase (BACE1), along with influential signaling programs of nuclear factor kappa-light-chain-enhancer of activated B cells (NF-*κ*B) and c-Jun N-terminal kinase (JNK), has helped visualize the path between OS and A*β* overproduction. In this review, attention will be paid to significant advances in the area of OS, epigenetics, and their influence on A*β* plaque assembly. Additionally, we aim to discuss available treatment options for AD that include antioxidant supplements, Asian traditional medicines, metal-protein-attenuating compounds, and histone modifying inhibitors.

## 1. Introduction

**Figure 1:**
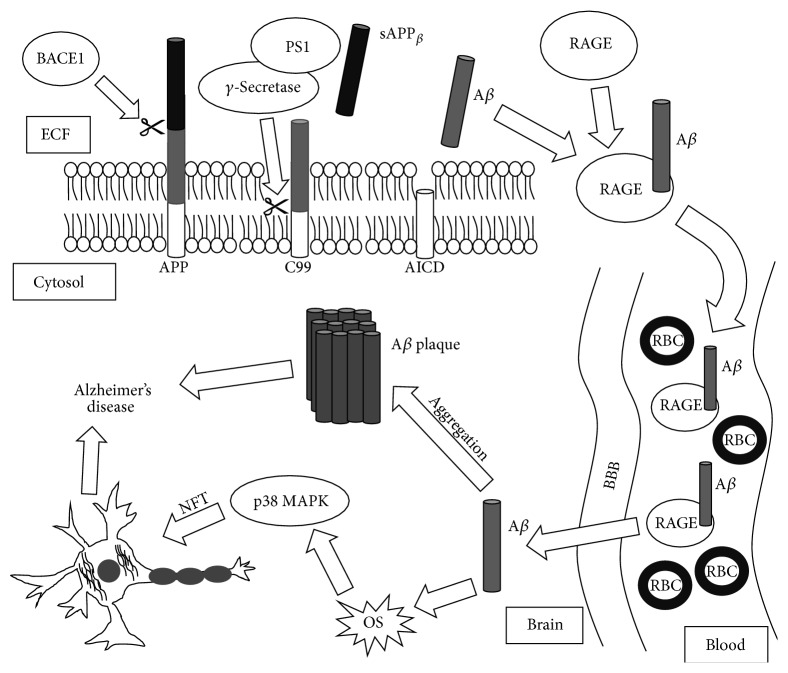
The role of amyloidogenesis in Alzheimer's disease. Amyloid-*β* originates as amyloid-beta precursor protein in the plasma membrane, which is cleaved sequentially by beta-site APP cleaving enzyme 1 and gamma-secretase. Following interaction between receptors for advanced glycation end products, which leads to its uptake in the brain, amyloid-*β* can form insoluble plaques and induce neurofibrillary tangles in neurons, the hallmarks of Alzheimer's disease. A*β*, amyloid-*β*; AICD, amyloid intracellular domain; APP, amyloid-beta precursor protein; BACE1, beta-site APP cleaving enzyme 1; BBB, blood-brain barrier; C99, residual 99 amino acids from C-terminal of APP; ECF, extra cellular fluid; p38 MAPK, p38 mitogen-activated protein kinase; OS, oxidative stress; PS1, presenilin 1; NFT, neurofibrillary tangle; RAGE, receptor for advanced glycation end products; RBC, red blood cell.

Alzheimer's disease (AD) is the most prevalent type of dementia with over five million people affected in the United States and 35 million worldwide [1]. The existence of amyloid-*β* (A*β*) plaques and tau neurofibrillary tangles (NFTs), leading to synaptic loss, is the major hallmark of AD pathogenesis [2–5]. A*β*, a 36–43 amino acid peptide that has been shown to clump together, forms amyloid aggregates that act in a prion fashion [6]. Zinc (Zn), Copper (Cu), and Iron (Fe) ions have also been implicated in the protein aggregation process, with Cu and Zn spatially correlated with amyloid plaques [7]. These plaques are often found in aging neurons, together with NFTs that are formed from hyperphosphorylated tau proteins. With time, this buildup of plaques and tangles can trigger the neurodegeneration associated with AD, resulting in cognitive deterioration with impaired speech, vision, behavior, and eventually death [8, 9]. Although individual facets of AD pathogenesis are understood, the mechanism of neurodegeneration is complex due to the fact that AD develops differently in each patient [10–12]. One possible vehicle for deposition and accumulation of A*β* in AD is oxidative stress (OS), mediated by the production of reactive oxygen species (ROS) (Figure 1) [3, 13–15].

Particularly in biological systems, ROS are a category of important free radicals such as superoxide and hydroxyl radicals produced as a byproduct of oxidative phosphorylation in the mitochondria's electron transport chain (ETC), with smaller amounts originating from cellular membrane, endoplasmic reticulum (ER), and peroxisomes [16–20]. Interestingly, A*β* can form complexes with Cu and generate hydrogen peroxide via the reduction of Cu^2+^ [21]. The body can be exposed to ROS directly from exogenous sources, such as tobacco smoke and radiation [22–24]. ROS can also act as necessary signaling molecules [25–27]. However, if their concentration increases markedly or the body cannot remove the excess species efficiently, these molecules can cause cellular damage. ROS oxidize essential cellular components such as DNA, proteins, and lipids, leading to disruption in cell homeostasis [28, 29].

In the process of aging and neurodegenerative diseases, the decline of normal antioxidant defense mechanisms increases the brain's vulnerability to oxidative damage known as OS [30]. ROS have the ability to modify crucial molecules in the cell, including proteins shown to be involved in neurodegenerative diseases [31]. Misregulation of ROS, due to either mitochondrial dysfunction, age, or both, has been implicated in numerous neurodegenerative diseases. By connecting ROS production to A*β* plaque formation, a more complete map of amyloidogenesis can allow researchers to focus their efforts on viable treatment options for AD.

## 2. Importance of ROS in Neurology

### 2.1. Natural Formation and Function of ROS

OS can be indicated by cell damage and impairment of cell signaling as a direct or indirect result of the accumulation of ROS in the cell. In some biological contexts, ROS provide essential molecular services. For example, neutrophils generate superoxide via NADPH oxidase in order to sequester or eliminate pathogens [32]. In many cases, superoxide forms from oxidative phosphorylation that occurs in the respiratory chain of mitochondria, especially in the sites of NADH dehydrogenase (complex I) and cytochrome bc1 (complex III) [13, 17]. The ETC transfers electrons from a series of protein complexes that act as electron donors and acceptors, with diatomic oxygen acting as the ultimate electron acceptor. Leakage in the ETC does occasionally occur, and premature redox reactions between oxygen and complexes I and III produce superoxide radicals [33]. Complexes I and III are also susceptible to malfunction during the process of aging, which can lead to additional OS [34]. ROS can be generated from pathological conditions such as hyperglycemia and hypoxic insults [3, 35, 36]. Aging is associated with increased OS due to long-term exposure of ROS and insufficient defense mechanisms in the brain [31, 34, 37]. The accumulation of such ROS eventually leads to significant cell damage [34, 38–41].

### 2.2. Inorganic-Related Pathoetiology

**Figure 2:**
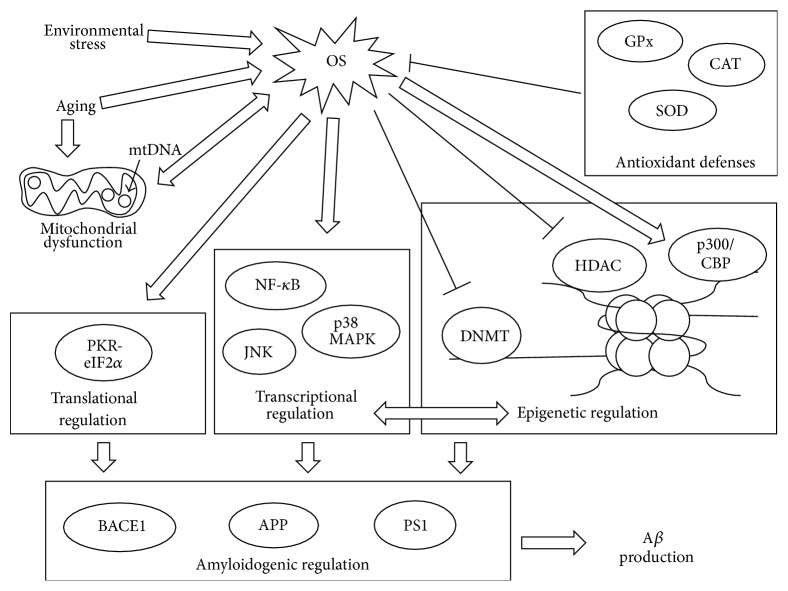
The role of oxidative stress in the multiple domains of amyloid-*β* production regulation. Oxidative stress, caused from reactive oxygen species production, creates an environment which is epigenetically, transcriptionally, and translationally favorable for amyloid-*β* production. A*β*, amyloid-*β*; APP, amyloid-beta precursor protein; BACE1, beta-site APP cleaving enzyme 1; CAT, catalase; DNMT, DNA methyltransferase; eIF2*α*, eukaryotic translation initiation factor-2alpha; GPx, glutathione peroxidase; HAT, histone acetyltransferase; HDAC, histone deacetylase complex; JNK, c-Jun N-terminal kinase; p38 MAPK, p38 mitogen-activated protein kinase; NF-*κ*B, nuclear factor kappa-light-chain-enhancer of activated B cells; OS, oxidative stress; PKR, double-stranded RNA dependent protein kinase; PS1, presenilin 1; SOD, superoxide dismutase.

Exposure to environmental factors such as pollutants, chemicals, and radiation can disrupt the balance between prooxidant and antioxidant levels, thereby inducing OS [5, 42]. Elevated ROS are due to activated phagocytes in chronic inflammation under stress, whereas the declining antioxidant levels are associated with mutated antioxidant enzymes or toxins [42]. Poisoning in herbicides, such as paraquat, can indirectly influence neurotransmitter metabolism by altering redox cycling [42] and has been linked to Parkinson's disease [43]. Nutritional factors also play crucial roles in AD development: excess Fe deposits can increase the formation of ROS while Zn can contribute to A*β* peptide aggregation [5, 44]. Recent studies show a detection of higher Fe concentration in AD patient brains, particularly in the area of the hippocampus and the parietal cortex. Fe-induced ROS can damage the cell membrane via lipid peroxidation and the subsequent neurotoxicity leads to potential cell death [5, 45]. Though present in high concentrations in A*β* aggregates, Fe has not been copurified with A*β*. On the other hand, Cu and Zn copurify with A*β* in human* postmortem* brains [46]. In a study by Chen et al., Zn addition was exclusively found to encourage protofibril formation. This process occurred without nucleation of A*β* oligomers [47]. Additionally, Cu has been found to form enzymatic complexes with A*β* that mirror the antioxidant superoxide dismutase (SOD). These A*β*·Cu^2+^ complexes have long been understood to directly generate hydrogen peroxide via Cu^2+^ reduction [48]. More recently, Mayes et al. demonstrated how A*β* fibrils, when bound to Cu^2+^, could convert hydrogen peroxide into hydroxyl radicals [49]. Although further investigation on the mechanism is necessary, environmental and nutritional-derived OS proposes a novel approach to therapeutic strategies in neurodegenerative diseases (Figure 2) [5].

### 2.3. Endogenous Antioxidant Defense Mechanisms

ROS molecules are natural byproducts of normal cellular biochemistry. In order to maintain homeostasis, the body has evolved several endogenous antioxidant molecules and enzymes to mitigate ROS-induced cytotoxicity [41]. Some of the better-studied antioxidant enzymes include SOD, catalase (CAT), and glutathione peroxidase (GPx) (Figure 2). Humans possess three types of SOD. SOD1 and SOD3, which contain Zn and Cu as cofactors, are located in the cytoplasm and extracellular space, respectively. SOD2 is located in the mitochondria and binds to manganese as a cofactor [50]. These metalloproteins can facilitate the dismutation of superoxide into oxygen and hydrogen peroxide [29, 50]. Peroxide, still a ROS molecule, is further processed by the antioxidant CAT. CAT is a nearly universal enzyme for organisms exposed to oxygen and catalyzes the decomposition of hydrogen peroxide into water and oxygen [51]. The selenium containing GPx restores oxidized membrane lipids [52], while also reducing hydrogen peroxide to water [53]. With the coordination of these antioxidants, a healthy cell can effectively control the potential dangers of ROS.

### 2.4. Dysfunction due to ROS Production and Neurodegeneration

While these antioxidants can help protect an organism from oxidative damage, they themselves can also be oxidized by ROS. Choi et al. examined the oxidative modifications that SOD1 could undergo in AD brains [54]. SOD1 was found to be oxidized and carbonylated in the brain, and its activity was markedly reduced in patients with AD. The downregulation of intracellular SOD is found to contribute to the acceleration of A*β* oligomerization and initiate early onset of cognitive impairment [55]. SOD1 was also observed in protein aggregates, implicating its role in AD pathogenesis [54]. Casado et al. and Ansari and Scheff both demonstrated reduced levels of SOD, CAT, and GPx in AD patient blood samples and human* postmortem* frontal cortex samples, respectively [56, 57]. Additionally, glutathione (GSH) redox cycling is reported to be essential in the brain's detoxification of ROS [41]. As the most abundant antioxidant, GSH acts in the first response to OS [58]. Reduced antioxidant capacity is a hallmark of AD, and the ensuing OS can lead to neurodegeneration. This oxidative imbalance illuminates the potential brain damage and cellular dysfunctions arising from OS [3, 41, 59]. If the level of ROS exceeds the protection of endogenous antioxidant pathways in persistence, cell death is likely and is almost universally implicated in neurodegeneration [38].

Le Bras et al. examined how increased ROS could activate cell-death machinery [60]. ROS are able to promote mitochondrial membrane permeability, releasing calcium (Ca^2+^). ROS can also discharge amplification factors of mitochondria-induced apoptosis, activate caspases, and induce DNA damage. By initiating these hallmarks of mitochondria-induced apoptosis, ROS have the capacity to trigger neuronal death. Furthermore, mitochondrial DNA (mtDNA) is another target of oxidation by ROS (Figure 2) [61]. Liu et al. showed how large sections of mtDNA were mutated in patients with neurodegenerative diseases and how mutations could make mitochondria more vulnerable to apoptosis [61]. In addition, Patten et al. determined how ROS affected apoptosis through other means. For example, ROS have been shown to stabilize p53 and activate c-Jun N-terminal kinase (JNK) [62]. Activation of these central elements in mitochondria-induced apoptosis can lead to eventual neurodegeneration.

### 2.5. Aging and Oxidative Stress

Many neurodegenerative diseases are associated with old age [63], and most symptoms appear in people over age 60 [64]. Recent research has suggested that the elderly are more prone to OS (Figure 2) [65, 66]. Complex I deficiencies are more prevalent in aging patients, suggesting that a less efficient ETC can create higher concentrations of ROS than in younger people [67]. Hamilton and Holscher used an AD mouse model to show that neurogenesis was markedly reduced in aging mice, together with increased A*β* plaque formation as a result of OS [65]. Additionally, mitochondria can be viewed as dynamic organelles, which are constantly undergoing a process of fusion and fission [68]. Conley et al. and Hauptmann et al. have demonstrated how mitochondrial dysfunction is more common with age [69, 70]. If the mitochondrial fission-fusion homeostasis is disrupted, accelerated ROS production will ensue (Figure 2) [71]. The resulting increase in ROS is detrimental for the cell due to superoxide and hydrogen peroxide's reactivity with essential molecules, including DNA and lipids [72]. Mitochondria accumulate membrane damage as they age, which can assist increased ROS production. Hauptmann et al. observed that mitochondrial dysfunction could begin as early as three months in an AD mouse model [70]. This is critical since mitochondrial dysfunction is generally viewed as one of the first steps of AD pathogenesis [37].

## 3. Neuropathological Characteristics of Alzheimer's Disease

### 3.1. Amyloidogenic Pathway

One of the major pathological indications of AD is the formation of extracellular plaques loaded with A*β* peptide [4, 73, 74]. Understanding the process in which A*β* is formed is likely of the utmost importance in the search for an effective AD treatment. The amyloid-beta precursor protein (APP) is an integral membrane protein whose normal function is not yet fully understood. By altering APP levels in transgenic mice, it was suggested that APP is important in synapse plasticity [75, 76]. APP is processed in two distinct mechanisms: the amyloidogenic, or plaque forming, and the nonamyloidogenic. In the nonamyloidogenic route, APP is processed by an *α*-secretase and then *γ*-secretase to yield an APP intracellular domain and soluble N-terminal fragment called p3 [77]. The majority of APP enters the nonamyloidogenic pathway, and other factors, such as mutations, environmental stimuli, and aging, are likely to influence this pattern; however, the mechanism remains unclear [4].

The sequential enzymatic breakdown of APP by the beta-site APP cleaving enzyme 1 (BACE1) and the *γ*-secretase complex with presenilin 1 (PS1) highlights the amyloidogenic pathway [2, 4, 5, 37, 78–80]. Other products formed from these actions include a truncated type of sAPP (sAPP_*β*_) and the residual 99 amino acids of APP (C99) from the cleavage of APP by BACE1. The remaining amyloid intracellular domain (AICD) is formed due to the liberation of A*β* cleaved from C99 by *γ*-secretase, leaving AICD in the plasma membrane (Figure 1) [81]. Though A*β* has observed beneficial characteristics, such as its function as an antimicrobial peptide [82], it is highly associated with formation of bulky plaques that ultimately result in neuronal degradation. Soluble A*β* oligomers are recognized as the most neurotoxic form of A*β* [41, 55, 83]. Its ability to bind to preexisting A*β* aggregates or lipid membranes (e.g., gangliosides) and potential to alter other cytoskeletal proteins can lead to synaptotoxicity within neurons [84]. Additionally, the activity of BACE1 is markedly higher in old age, linking age with A*β* plaque production [85]. The receptor for advanced glycation end products (RAGE) is an important A*β* receptor and the binding of A*β* to RAGE facilitates transportation across the blood-brain barrier (BBB) [86], thereby aiding in accumulation of A*β* protein within the brain (Figure 1). Cho et al. described how RAGE could stimulate BACE1 expression through RAGE's ability to generate an intracellular Ca^2+^ response that activates nuclear factor of activated T-cells 1 (NFAT1), a BACE1 activator. BACE1 then cleaves APP to form A*β*, which completes the feedback loop by acting as a RAGE activator [87].

### 3.2. Connection between A*β*-Induced OS and Tau Neurofibrillary Tangles (NFTs)

Another pathophysiological characteristic of AD is the formation of intracellular NFTs consisting of an abnormal accumulation of hyperphosphorylated tau protein [4, 73]. Normally, tau serves to assemble and stabilize microtubule structures and is essential for the transportation of vesicles containing neurotransmitters within the neuronal axons. The excess phosphorylated tau aggregates and forms insoluble helical filaments that limit the transportation of neurotransmitters. As a result, NFTs interfere with communication between neurons and eventually lead to cognition impairment. Like A*β* oligomers, tau aggregates are cytotoxic [4].

A*β*-induced OS alters cellular signaling pathways and initiates a phosphorylation response. An increase in activation of JNK and p38 mitogen-activated protein kinase (MAPK) is noticeable in AD* postmortem* brains, suggesting a possible linkage between dysregulation of MAPK signaling pathway and AD pathogenesis [88]. Giraldo et al. demonstrated that p38 MAPK polypeptide is involved in tau hyperphosphorylation. The p38 MAPK and other kinases can be activated in response to A*β* accumulation. Activated p38 MAPK polypeptide phosphorylates tau protein, producing a hyperphosphorylated tau response. This study illustrates a positive correlation between tau aggregation and activated p38. Therefore, the activation of p38 is an indicator for tau hyperphosphorylation, further supporting the pathological association between A*β* and tau in AD (Figure 1) [88].

### 3.3. A*β* Formation Leads to Apoptosis in Neurons

A*β* has been associated with neurodegeneration and is found at elevated levels in AD brains [89]. The increase in A*β* causes neurodegeneration by activating apoptotic death signals such as caspase pathways in neurons [90–93]. Ferreiro et al. stated that A*β* was involved in depleting Ca^2+^ amounts in the ER [90], resulting in a high level of cytosolic Ca^2+^ that causes the mitochondrial membrane to lose its chemical potential, inducing mitochondrial apoptotic events. They demonstrated that lower levels of GSH, in response to increased Ca^2+^ release, result in ROS accumulation [91]. A*β* has also been shown to increase the activity of calcineurin (CaN), a protein phosphatase that catalyzes dephosphorylation of Bcl-2-associated death promoter (BAD). As a proapoptotic protein, BAD triggers cytochrome c release after translocating to the mitochondria [92]. In addition, A*β* proteins associate with the caspase cascade, leading to proteolysis of caspase targets and eventual apoptosis [93].

### 3.4. Neurodegeneration Results in Decreased Cognitive Ability, Dementia, and Memory Loss

A*β* has been referred to as an initiator in the mitochondrial, ER, and caspase-responsive apoptotic pathways, which collectively lead to neurodegeneration [90–93]. Neuronal atrophy is an essential characteristic of AD, as well as memory deficits, a loss of cognitive ability, and dementia [8, 94]. A study of 764 participants attempted to map A*β* in the brain. Posterior cortical regions, associated with memory retrieval in younger participants, show A*β* deposits in the elderly with AD [95]. Aging mice expressing an AD-linked APP variant formed A*β* plaques, resulting in memory loss [96]. The isolated A*β* protein induced memory deficits when injected into young rats [96]. Similarly, A*β* dimers extracted from the cerebral cortex of AD patients were found to affect learned behavior memory when administered to normal rats. The A*β* dimers were concluded to be the smallest toxic species for synapse structure [97]. Therefore, A*β* has been reliably shown to induce AD effects in a variety of experimental settings.

## 4. The Role of ROS in Alzheimer's Disease

### 4.1. Epigenetic Alteration of A*β* (Methylation and Acetylation)

ROS activity has long been understood to affect DNA transcription through its oxidation of DNA and related proteins [98, 99]. Epigenetics refers to the changes in gene expression through chemical processes, such as histone modification and DNA methylation, without the disruption of DNA sequence. Acting as an anchor for DNA, histones contain N-terminal tails that can be methylated, sumoylated, phosphorylated, and ubiquitinated, as well as other posttranslational modifications. Histone acetylation and deacetylation are the most well-studied mechanisms, with histone acetyltransferases (HATs) and histone deacetylases (HDACs) attaching or removing acetyl groups to the histone tails, respectively. Acetylation neutralizes the positive charge associated with the histone tail, limiting the attraction among the negative phosphate groups of DNA. Relaxed DNA offers easier access for gene transcription [100]. DNA methyltransferases (DNMTs) are closely tied to the process of histone acetylation, modifying DNA structure by transferring methyl groups to cytosine-guanine (CpG) dinucleotides. Generally, methylated CpG sequences can induce histone modifications that repress the transcriptional complex's ability to access DNA [100]. Oxidation of the guanine residue in CpG dinucleotides can also affect the epigenetic regulatory complexes in a similar manner, placing emphasis on OS in the regulation of CpG sites [101]. These sites are particularly important in AD, as the promoter regions of APP and BACE1 contain 65 and 36 CpG sites, respectively [3]. The presence of these sites adds significance to the idea that the essential genes of amyloidosis are potentially regulated in an epigenetic manner. Additionally, epigenetics can be influenced by environmental stimuli; however, it can also change naturally during growth and development [102].

Recently, explorations of epigenetic regulation mechanisms present a novel insight into OS and its relation to AD [3, 103]. Several studies have revealed the epigenetic control of A*β* production in the progression of AD [104, 105]. Chromatin remodeling has also been reported to assist in the upregulation of BACE1 and A*β* production [106, 107]. Sung et al. and Chouliaras et al. have shown that not only is there global decrease of DNA methylation in the hippocampus of* postmortem* AD patients, but also APP-related mutations cause an epigenetic shift in an AD model cell line [102, 103]. Clearly, epigenetic mechanisms are meaningful in A*β* plaque formation. Gu et al. studied what possible agents could provoke this epigenetic shift in AD patients [3]. When neuroblastoma cells were treated with hydrogen peroxide, there was a significant increase in histone acetylation together with a decrease in DNA methylation. This histone hyperacetylation and DNA hypomethylation resulted in increased APP and BACE1 transcription, possibly by a gain of nuclear factor kappa-light-chain-enhancer of activated B cells (NF-*κ*B) activity [3]. This study shows how OS can cause A*β* plaque formation through means of epigenetic mechanisms and offers promise for treatment approaches directed at this pathway. Cytosine in a CpG site of DNA undergoes frequent methylation and regulates gene expression during development, differentiation, pathogenesis, and aging [3, 102]. Besides DNA methylation, DNA hydroxymethylation describes a different biological role in the epigenetic modification of AD. 5-Hydroxymethylcytidine (5-hmC) and 5-methylcytidine (5-mC) levels are significantly decreased in AD brains [103]. The linkage between epigenetic dysregulation and AD is evidently supported by previous studies with correlation to OS [3, 103]. The modified transcriptional expression of AD-related genes (APP, BACE1, and PS1) enhances A*β* production and contributes to AD pathogenesis and development [3, 108]. Furthermore, epigenetic mechanisms associated with OS, especially altered methylation or CpG oxidation, exacerbate the progression of oxidative DNA damage (Figure 2) [3].

### 4.2. Activation of Stress-Related Signal Pathway Increases BACE1 and APP Transcription

Due to the neuropathological nature of AD, many studies have investigated how the A*β* formation pathway can be manipulated. Using the potent DNMT inhibitor S-adenosylhomocysteine (SAH), Lin et al. were able to hypomethylate PS1 and APP promoters, accompanied by increased expression of PS1 and APP. As a result, A*β* protein production is increased [109]. Guo et al. showed that JNK and p38 MAPK, stress-related MAPKs, are activated with addition of anisomycin and induce intracellular A*β* production in neuroblastoma cells [110]. APP and BACE1 were found to be upregulated as a result of demethylation of their promoters. Simultaneously, transcription of HAT p300/CREB-binding protein (CBP) was increased, while transcription of DNMTs and HDACs was downregulated [110]. This study confirmed that A*β* overproduction could occur during times of cellular stress through a hypomethylation/hyperacetylation-dependent pathway. Increasingly, evidence suggests the contribution of epigenetic dysregulation to AD pathogenesis [25, 102]. The epigenetic modification in AD-related genes is a result of activation of JNK and p38 MAPK pathways [3, 111]. In the presence of anisomycin, the reduction of DNMT activity induces an overexpression of APP, BACE1, and PS1 [102, 112]. In addition, Gu et al. have identified hydrogen peroxide as an activator for the distinct MAPK cascades. DNA methylation is markedly reduced in APP and BACE1 promoters after treatments with hydrogen peroxide [3]. The finding further suggests the role of OS in modulating DNA methylation and histone acetylation in specific AD-related genes (Figure 2) [3, 25].

### 4.3. Translational Regulation of A*β*

In a separate investigation, the addition of hydrogen peroxide in human neuroblastoma cells resulted in enhanced expression of BACE1, supporting the observation that OS can heighten BACE1 levels [111]. JNK is believed to be responsible for this increase in BACE1 mRNA, while JNK signaling is correlated with tau-induced toxicity [38]. Moreover, eukaryotic translation initiation factor-2alpha (eIF2*α*) was found to translationally regulate initiation of BACE1 protein synthesis [111]. The eIF2*α* undergoes phosphorylation upon its activation, and elevated levels of phosphorylated eIF2*α* have been reported in AD brains [113]. Phosphorylated eIF2*α* generally stops protein translation in response to cellular stress; however certain stress response genes, such as BACE1, are activated by eIF2*α* [111]. Double-stranded RNA dependent protein kinase (PKR) responds to cellular hardship in a proapoptotic manner by activating other stress signaling cascades including eIF2*α* [114]. Therefore, PKR-eIF2*α* expands BACE1 protein expression via translational regulation in response to OS (Figure 2) [111]. Suppression of aberrant eIF2*α* phosphorylation ameliorated AD symptoms in a mouse AD model [113].

## 5. Treatment

### 5.1. Antioxidant Supplements

Promising strategies for AD treatment fall on those that can decrease A*β* oligomer and phosphorylated tau levels, promote neuroprotection, and alleviate OS [112, 115]. With the view that ROS are the instigators in A*β* production, it is understandable that much research has focused on the clinical opportunity of antioxidants in alleviating AD symptoms. Gubandru et al. measured the effects of several antioxidant supplements on certain OS markers such as advanced glycation end products (AGEs), protein carbonyls (CRBNLs), and malondialdehyde (MDA) [73]. Although the sample size was small (21 AD patients and 10 controls), results demonstrated how the antioxidant supplement Rivastigmine decreases AGEs in AD patients, while Donepezil restores GSH and total antioxidant capacity (TAC). Therefore, antioxidant supplements offer potential strategies to ameliorate AD in dementia patients [73].

JNK and NF-*κ*B are well-known activators of the amyloidogenic pathway and are responsive to OS [116, 117]. As a free radical scavenger, molecular hydrogen (H_2_) can specifically reduce hydroxyl radicals. To that end, Wang et al. demonstrated how the reducing agent hydrogen-rich saline could decrease neural inflammation and OS induced by A*β* [118]. Specifically, mice were injected with A*β* and then treated with hydrogen-rich saline for 10 days. OS markers, including the levels of 8-hydroxydeoxyguanosine (8-OHdG), JNK, and NF-*κ*B, were all reduced in the hydrogen-rich saline administered group. Therefore, hydrogen-rich saline inversely regulates the activation of JNK [118]. This study suggests that hydrogen-rich saline can be used to relieve the symptoms of neuroinflammation [119] and OS in AD patients by attenuation of JNK and NF-*κ*B-induced OS response [118].

Rutin is a naturally occurring glycoside that acts as both an anti-inflammatory and an antioxidant agent. Wang et al. previously demonstrated rutin's ability to inhibit A*β* plaque formation and relieve OS [120]. In another study directly aimed at rutin's role in protecting against AD, mice treated with rutin displayed favorable levels of antioxidant markers, such as increased SOD and GPx activity, reduced memory deficits, and fewer A*β* oligomers [41]. Inflammatory cytokines interleukin- (IL-) 1*β* and IL-6 were also found to be at lower levels in treated murine brains. In addition, rutin supplementation enhances the activity of SOD, GPx, and CAT [121]. Due to its demonstrated antioxidant and anti-inflammatory properties, rutin shows great potential as a future treatment for AD patients.

Resveratrol, normally found in grapes and red wine, is a phytoalexin that is produced by plant species as a defense mechanism against fungal attack. Its neuroprotective/antioxidant properties have been shown useful in AD treatment [94]. For example, resveratrol can protect astrocytes, the human brain's most plentiful cell, against ROS damage. Astrocytes are important sources of GSH, a major antioxidant in the body, and a decline in GSH levels occurs in aged brains due to the increased vulnerability against OS [122]. Resveratrol provides a shield for astrocytes, which in turn modulates GSH levels and reinforces its antioxidant activity [122]. In addition, resveratrol hinders cellular apoptosis through influencing intracellular signaling pathways and antioxidant mechanisms, such as reducing NF-*κ*B activation and scavenging ROS intermediates. Particularly, resveratrol activates sirtuin protein, a NAD-dependent HDAC, and ultimately improves mitochondrial bioenergetic efficiency through a pathway mediated by sirtuin-1 (SIRT1) and inhibits the formation of A*β* fibrils [94, 123]. Thus, resveratrol's potential to protect neurons from A*β*- and OS-induced toxicity displays promising therapeutic applications during AD progression.

Vitamin E includes a group of antioxidant molecules called tocopherols and tocotrienols [124]. Given its free radical scavenging activity and biological significance in treating other diseases, Vitamin E as a therapy for AD has attracted much attention. Interestingly, a study examining the effect of Vitamins A, C, and E for OS concluded that not only could Vitamin E restore antioxidant activity, but it was found to be more effective than Vitamin A and C, in the rat brain [125]. However, in a more recent study there was no significant difference recorded between patients taking Vitamin E (800 IU/day) over those taking a placebo [126]. A review of the effect of Vitamin E in AD treatment highlights the conflicting results of many studies [127]. Mecocci and Polidori concluded that various obstacles, such as the permeability of the BBB, the delicate antioxidant/free radical equilibrium, and vascular factors of AD pathogenesis, could be responsible for reduced Vitamin E efficacy [128]. Focusing on improving Vitamin E uptake in the brain and optimizing a treatment plan should lead to more realistic results for Vitamin E supplementation. These challenges make Vitamin E and other antioxidants difficult but worthy potential AD treatment candidates [41, 120, 122, 128].

### 5.2. Traditional Medicine

Traditional Chinese and Ayurvedic medicine has led to various potential candidates for AD treatment. Su He Xiang Wan (SHXW) is a combination of herbs used in traditional Chinese medicine for clinical problems including stroke, infantile convulsions, and seizures [129, 130]. Jeon et al. studied the effect of SHXW when inhaled by mice injected with A*β* into the hippocampus [130]. SHXW mice showed reduced memory impairment and suppressed A*β*-induced JNK and p38 activations. In SH-SY5Y cells, repression of A*β*-induced apoptosis was observed, and upregulation of Heme oxygenase (HO-1) and nuclear factor-like 2 (Nrf2) indicated mitigated ROS formation [130]. Together, these findings suggest the promise of SHXW to treat AD patients. Clinical studies remain to be conducted to determine the potential efficacy of SHXW.

The Chinese native* Ginkgo biloba* tree has a long history of practice in Chinese traditional medicine [131]. Investigation of its potential role in western medicine has yielded mixed results. A study was performed on 395 subjects with dementia who were treated with 240 mg/day of EGb 761, an extract from* Ginkgo biloba* leaves. Patients that were treated with EGb 761 scored higher on the Short Syndrome Test (SKT), a measure of cognitive ability [132]. A second study showed similar results on the SKT, as well as improved secondary efficacy variables such as caregiver distress scores, Alzheimer's Disease Activities of Daily Living International Scale, and Verbal Fluency Test [133]. However, a separate long-term study testing the effectiveness of EGb 761 in the prevention of AD showed no significant difference between EGb 761 and the matched placebo in terms of AD diagnosis [134], which requires further investigation.

In India, the spice turmeric is a major constituent in curry powders and has been used for digestive ailments [135]. Curcumin, a molecule found in turmeric, has antioxidant activity [136]. Lim et al. observed the effect of low (160 ppm) and high (5000 ppm) dose curcumin on Alzheimer-like pathology in mice. Both high and low concentrations led to a reduced amount of oxidized proteins, as indicated by western blot analysis of carbonylation levels in murine brains. In addition, low dose curcumin lowered soluble and insoluble A*β* by up to 50%. However, APP expression remained unchanged [136]. Yang et al. demonstrated that curcumin can cross the BBB to bind to A*β* and hinder aggregation of A*β*, reducing A*β* levels in mice with previously established A*β* deposits [137].

### 5.3. Metal Ion Chelators

As mentioned earlier, Fe, Cu, and Zn have been implicated in various aspects of AD, including Fe-induced cognitive damage [45], Cu- and Zn-mediated amyloid aggregation [138], and Cu-mediated ROS generation [48, 49]. Investigations into the possibility of metal-protein-attenuating compounds (MPACs) that abate proteins from interacting with ions have yielded promising results. Iodochlorhydroxyquin, commonly known as clioquinol, is an MPAC that was the focus of a pilot phase 2 clinical trial carried out by Ritchie et al. Over 9 months, 36 patients participated in a double-blinded, placebo-controlled study that showed a reduction in plasma A*β*42 levels, with no changes in Cu levels. Although the myelo-optic neuropathy associated with chronic use of clioquinol caused its withdrawal in 1970, clioquinol appears to be safe to use with no adverse effects that were reported in this study [139]. Separately, Lannfelt et al. examined the efficacy of PBT2. A successor to clioquinol, PBT2 is a second-generation 8-OH quinolone MPAC that also demonstrated beneficial effects in targeting A*β* oligomers. A*β*42 levels were lowered dose-dependently of PBT2, and no severe adverse effects were reported [140]. Further research into the efficacy and safety of MPACs could hold much potential in the search for effective AD treatment.

### 5.4. HDAC Inhibitors

Epigenetically, AD genomes have been found to be globally DNA-hypomethylated and histone-hyperacetylated [141]. This epigenetic profile is beneficial for BACE1 expression and A*β* production, thus leading to AD formation. If these epigenetic changes could be reversed, possibly A*β* aggregation could be suppressed. To test this idea, Sung et al. developed two novel HDAC inhibitors (HDACIs) to determine A*β* levels in response to histone deacetylation [108]. Overall, A*β*40 and A*β*42 levels, two common sizes of the A*β* protein, were lower with exposure to HDACIs* in vitro*. *β*- and *γ*-Secretase component transcription was suppressed and transcription of A*β* degrading enzymes, such as matrix metalloproteinase-2 (Mmp2), was increased. Additionally, aged AD mice showed improved learning capabilities and reduced memory deficits when exposed to HDACIs [142]. Addition of curcumin, a p300 inhibitor, reduced the expression of BACE1 via histone H3 acetylation inhibition in an Alzheimer cell line [143], further promoting the idea of epigenetics as an initial step in A*β* production. Thus, these inhibitors can be potentially used as alternative treatment options for AD in clinical settings.

## 6. Conclusions

The features of AD pathogenesis are interrelated with OS. Although it remains unclear whether OS is a direct cause or a result of AD pathology, evidence demonstrates that A*β* plaques, NFTs, and mitochondrial dysfunction all contribute to and are influenced by the imbalance of the oxidative state in the brain. Overproduction of A*β* protein is increased through upregulation of both APP and BACE1, as well as involvement of transcriptional and translational coordinators. The activation of stress-induced MAPK (e.g., p38 MAPK) signaling pathways further contributes to the hallmarks of AD. Therapies that include a diet with high levels of antioxidants could both guard against deleterious epigenetic changes and alleviate the devastating clinical manifestations of AD. Additionally, compounds derived from traditional Chinese and Ayurvedic medicine could potentially be candidates for clinical trials given their success in the laboratory. MPACs that target the impact of metal ions in OS and protein aggregation, as well as inhibitors of the HAT/HDAC enzymes, restore global epigenetic expression that is altered by OS. Inhibition of this event reduces apoptosis and neurodegeneration observed in histone-altered cells. Studies that continue to elucidate the exact mechanism of OS-induced A*β* production and the effectiveness of antioxidants and small molecule inhibitors will be paramount to the treatment of AD. With increasing understanding of AD pathogenesis, the findings provide promising prospects guiding future clinical investigations and discovery of novel treatment approaches.
